# Clinical manifestations and immune correlations in anti-centromere antibody-positive and anti-SSA/Ro antibody-positive primary Sjögren’s syndrome: A retrospective analysis

**DOI:** 10.1371/journal.pone.0322845

**Published:** 2025-08-15

**Authors:** Songyan Zou, Riyi Zhang, Dongdong Yang, Xiaodong Li, Naixiang Huang, Fuyi Xie, Yinyu Mu

**Affiliations:** Department of Clinical Laboratory, The Affiliated Lihuili Hospital of Ningbo University, Ningbo, Zhejiang, China; University Hospital of Bologna Sant'Orsola-Malpighi Polyclinic Department of Digestive System: Azienda Ospedaliero-Universitaria di Bologna Policlinico Sant'Orsola-Malpighi Dipartimento dell'apparato digerente, ITALY

## Abstract

Primary Sjögren’s syndrome (pSS) is an autoimmune disorder characterized by dry eyes and mouth, often with systemic involvement. This study aimed to investigate the clinical manifestations and laboratory features in pSS patients, particularly focusing on those with anti-centromere antibodies (ACA) and anti-SSA/Ro antibodies. We conducted a retrospective analysis of 152 patients diagnosed with pSS at Ningbo Medical Center Lihuili Hospital from 2018 to 2023, using the 2016 ACR/EULAR criteria. Additionally, 105 age- and sex-matched healthy controls were included to establish a comparative baseline for laboratory parameters. Clinical data were obtained from medical records, and patients were categorized into ACA-positive and SSA-positive groups based on their autoantibody profiles. Appropriate statistical analyses, including ANOVA, chi-square tests, correlation analysis, and multivariate regression analysis, were used to compare clinical and laboratory parameters in pSS patients. ACA-positive patients were significantly older and exhibited a higher prevalence of Raynaud’s phenomenon and left ventricular diastolic dysfunction, while SSA-positive patients presented with more marked hematological abnormalities. ACA-positive patients have a more prevalent occurrence of positive antinuclear antibodies (ANA) and anti-mitochondrial M2 antibodies (AMA-M2), as well as significantly higher levels of left ventricular end-diastolic diameter (LVEDD), IgM, and lactate dehydrogenase (LDH). In addition, SSA-positive patients exhibited elevated levels of globulins (GLB), IgG, IgA, and rheumatoid factor (RF), accompanied by decreased albumin (ALB) levels. ACA-positive patients had abnormal proportions of CD4^+^ and CD8^+^ T cells and had reduced counts of NK cells (CD16^+^CD56^+^), CD3^+^ T cells, and CD8^+^ T cells. Correlation and multivariate regression analyses revealed a significant association between NK cell and levels of IgG, IgM, GLB, and LDH. Furthermore, SSA-positive patients showed abnormal proportions of CD19^+^ B cells and NK cells, with reduced counts of CD3^+^ T cells (including CD4^+^ and CD8^+^ T cells) and NK cells. Correlation and multivariate regression analyses indicated a significant correlation between CD4^+^ T cell and levels of IgG, ALB, and GLB. Overall, T cell-mediated immunity plays a significant role in SSA-positive patients; NK cells are shown to be critically involved in the pathogenesis of ACA-positive pSS.

## Introduction

Primary Sjögren’s syndrome (pSS) is a chronic inflammatory autoimmune disorder marked by the progressive infiltration of immune cells into exocrine glands, which results in gland dysfunction and manifests as dryness in the affected areas [1]. Beyond dry symptoms, pSS is also associated with systemic manifestations, including fatigue, arthralgia, and involvement of other organs such as the kidneys, liver, heart, and lungs. The variable presentation of this condition, with differing degrees of glandular and extraglandular involvement, poses challenges for diagnosis and treatment [2,3]. A comprehensive understanding of the underlying mechanisms, clinical presentations, and laboratory characteristics of pSS is vital for effective patient management and the development of therapeutic strategies.

The presence of specific autoantibodies plays a crucial role in the diagnosis, disease stratification, and treatment decision-making for pSS patients. Two of the most significant autoantibodies found in pSS patients are anti-SSA/Ro antibodies and anti-centromere antibodies (ACA) [4,5]. Studies indicate that anti-SSA/Ro antibodies can be detected in approximately 33–74% of pSS patients, while anti-centromere antibodies are found in a smaller subset, typically ranging from 4–27% [1,6,7]. The ACA was initially identified in patients with limited cutaneous systemic sclerosis. Studies have shown that ACA is not only significantly associated with the clinical phenotype of pSS but also correlates with other autoimmune diseases, such as systemic sclerosis (SSc) [7,8]. It has been reported that ACA-positive pSS patients differ from ACA-negative patients in terms of clinical and laboratory features, including an older age at diagnosis, more frequent occurrences of Raynaud’s phenomenon, liver damage, objective symptoms of dry eyes, peripheral neuropathy, lower prevalence of rheumatoid factor, and a lower incidence of leukopenia and hypergammaglobulinemia [8–10]. These studies indicate that ACA-positive patients represent a distinct subgroup of pSS, characterized by specific clinical manifestations, disease course, and prognosis. However, there are limitations and controversies surrounding certain outcomes associated with ACA positivity. Distinguishing the clinical and laboratory findings associated with the two subtypes of pSS is key to unraveling the disease’s pathogenesis and to the customization of treatment plans.

This study focuses on the clinical manifestations and laboratory features in pSS patients, specifically those positive for anti-centromere antibodies and anti-SSA/Ro antibodies. The research aims to clarify the differences in clinical manifestations between these two groups of pSS patients, as well as to explore the correlation between laboratory indicators and these clinical features.

## Materials and methods

### Study design and patients

This study is a single-center retrospective analysis utilizing electronic health records of patients diagnosed with pSS who were hospitalized at Ningbo Medical Center Lihuili Hospital between January 2018 and December 2023. Inclusion criteria were as follows: 1) patients met the diagnostic criteria for pSS established by the 2016 ACR/EULAR guidelines [11]; 2) patients were over 18 years of age; 3) patients were newly diagnosed and had not used glucocorticoids or immunosuppressants in the past three months. Exclusion criteria included: 1) patients with concomitant conditions such as systemic lupus erythematosus (SLE), rheumatoid arthritis (RA), systemic sclerosis (SSc) or mixed connective tissue disease (MCTD); 2) a history of radiation therapy to the head and neck, hepatitis C virus infection, sarcoidosis, amyloidosis, AIDS, graft-versus-host disease, or IgG4-related diseases; 3) pregnant or breastfeeding women; 4) patients with incomplete clinical data. Ultimately, 152 patients were included in the study. Additionally, 105 age- and sex-matched healthy individuals who underwent physical examinations at the same hospital during the same period were selected as healthy controls. Based on their autoantibodies, the pSS patients were divided into an ACA-positive group (n = 53) and an SSA-positive group (n = 99). The ACA-positive group had only anti-centromere antibodies, while the SSA-positive group exclusively had anti-SSA/Ro antibodies. This study was approved by the Ethics Committee of Ningbo Medical Center Lihuili Hospital (Ethics Approval No.: KY2024SL337–01), and the requirement for written informed consent was waived due to the retrospective nature of the study. Data were accessed for research purposes from October 25, 2024, to November 4, 2024.

### Data collection

Demographic and clinical variables, including gender, age, disease duration, extra-glandular manifestations, and comorbidities, were extracted from the electronic medical records system of Ningbo Medical Center Lihuili Hospital. Systemic involvement was defined according to the 2010 EULAR Sjögren’s Syndrome Disease Activity Index (ESSDAI) [12]. Articular involvement was characterized by arthralgias with morning stiffness lasting over 30 minutes or joint synovitis. Cutaneous involvement was defined as cutaneous vasculitis including urticarial vasculitis, diffuse purpura, ulcers related to vasculitis and so on. Neurological involvement referred to damage to the peripheral and/or central nervous systems. Pulmonary involvement was defined as pulmonary fibrosis or interstitial lung disease (ILD) accompanied by respiratory symptoms or abnormal pulmonary function tests. Renal involvement included renal tubular acidosis (with or without renal failure) or glomerular involvement with proteinuria exceeding 0.5 g/day. Digestive involvement encompassed impaired digestive function, autoimmune hepatitis (AIH), autoimmune cholangitis, or primary biliary cholangitis (PBC). Hematological involvement was defined as leukopenia, anemia, lymphopenia, or thrombocytopenia after excluding medication effects or other diseases. Additionally, sclerodactyly, characterized by thickening and hardening of the skin of the fingers, was recorded. Left ventricular diastolic dysfunction was assessed by two experienced sonographers using the mitral E/A ratio and/or the septal basal E’/A’ ratio, with a diagnostic threshold below 1 indicating compromised myocardial relaxation [13]. Electrocardiographic abnormalities included atrioventricular conduction block, non-specific ST-segment changes, abnormal T waves, axis deviation, bundle branch block, and prolonged QT interval. Autoimmune thyroid diseases included Graves’ disease and Hashimoto’s thyroiditis.

Laboratory parameters, including biochemical indicators, antinuclear antibodies (ANAs), autoantibodies, immunoglobulins, complements, rheumatoid factors, routine blood tests, lymphocyte subsets, and echocardiographic assessments, were obtained by reviewing the medical records. Biochemical indicators, such as alanine aminotransferase (ALT), aspartate aminotransferase (AST), gamma-glutamyl transpeptidase (GGT), alkaline phosphatase (ALP), and lactate dehydrogenase (LDH), were measured using the velocity method on an automatic biochemical analyzer (ADVIA2400, Siemens Healthineers, Germany). Total protein (TP) and albumin (ALB) levels were determined via the biuret method and bromcresol green method, respectively, while globulin (GLB) levels were calculated by subtracting albumin from total protein. ANAs were detected using an indirect immunofluorescence assay (IIFA) with a commercial kit (Euroimmun AG, Lübeck, Germany), with HEp-2 cells as the substrate. Autoantibodies against nRNP, Sm, SSA/Ro60, SSA/Ro52, SSB/La, Scl-70, PM-Scl, Jo-1, PCNA, CENP-B, AMA M2, dsDNA, histones, nucleosomes, and RibP were detected by immunoblotting (Shenzhen YHLO Biotech Co., Ltd, China). Immunoglobulins (IgG, IgM, IgA), complements (C3, C4), and rheumatoid factors (RF) were quantitatively assessed by immunonephelometry (IMMAGE 800, Beckman Coulter, USA). Routine blood tests, including white blood cells (WBC), neutrophils (NE), lymphocytes (LC), monocytes (Mono), red blood cells (RBC), hemoglobin (Hb), and platelets (PLT), were measured using flow cytometry (XN-2800, Sysmex Corporation, Japan). Lymphocyte subsets (CD3^+^, CD3^+^CD4^+^, CD3^+^CD8^+^, CD19^+^, CD16^+^CD56^+^) were analyzed by flow cytometric immunophenotyping (FACS Canto, Becton Dickinson, USA) with FlowJo Software (Treestar, USA). Echocardiographic assessments were performed using the Philips IE 33 color Doppler ultrasound system (Philips Healthcare, USA), with measurements including left ventricular end-diastolic diameter (LVEDD), interventricular septum thickness (IVST), left atrial diameter (LAD), and left ventricular ejection fraction (LVEF).

### Statistical analysis

Data analysis was performed using SPSS 27.0 (IBM Corp., Armonk, N.Y., USA) and GraphPad Prism 9 software (GraphPad Software Inc., La Jolla, California, USA). The normality of the data was assessed with the Shapiro-Wilk test, and variance homogeneity was evaluated using Levene’s test. Continuous data conforming to a normal distribution are presented as mean ± standard deviation. For datasets with homogeneous variance, ANOVA was used for comparisons among multiple groups, with the Scheffé method employed for post-hoc multiple comparisons. For datasets with heterogeneous variance, Welch’s ANOVA was utilized, followed by Games-Howell post-hoc comparisons. Skewed data are presented as median (Q1, Q3); comparisons between ACA-positive and SSA-positive groups were performed using the Mann-Whitney U test, and Kruskal-Wallis test was utilized for comparisons among other multiple groups, with post-hoc assessments adjusted using the Bonferroni correction for multiple comparisons. Categorical data are expressed as n (%), with group comparisons assessed using Chi-squared tests or Fisher’s exact test as appropriate. Post-hoc comparisons were conducted with Bonferroni correction to adjust for multiple testing. Pearson or Spearman rank correlation analysis was used to examine the relationships between immune markers, biochemical parameters and lymphocyte subsets. The relationship between immune markers, biochemical parameters and lymphocyte subsets was analyzed using multivariate regression analysis. A P-value <0.05 (two-tailed) was considered statistically significant.

## Results

### Demographic characteristics and clinical manifestations

The demographic and clinical features of the three groups are presented in Table 1. There were no significant differences in gender distribution among the groups. However, ACA-positive pSS patients exhibited a significantly older age compared to SSA-positive pSS patients (P < 0.05). The ACA-positive group showed a significantly higher prevalence of Raynaud’s phenomenon (16.98%) and left ventricular diastolic dysfunction (50.94%) than the SSA-positive group (3.03% and 33.33%, respectively; P < 0.01 for Raynaud’s phenomenon and P < 0.05 for left ventricular diastolic dysfunction). Conversely, systemic involvement of the hematological system was more pronounced in the SSA-positive group, with significant differences in anemia (29.29% vs. 9.43%, P < 0.01), leukopenia (31.31% vs. 13.21%, P < 0.05), and neutropenia (22.22% vs. 7.55%, P < 0.05). There were no significant differences in the involvement of the skin, joints, digestive system, lungs, kidneys, or nervous system between the two groups. Similarly, no significant differences were observed in the history of hypertension, diabetes, malignant tumors, or the presence of sclerodactyly, electrocardiographic abnormalities and other autoimmune diseases.

**Table 1:** Demographics and clinical features of healthy controls and primary Sjögren’s Syndrome patients.

Parameters	Healthy controls (n = 105)	ACA-positive (n = 53)	SSA-positive (n = 99)	P-value
**Female sex, n (%)**	95 (90.48)	51 (96.23)	88 (88.89)	0.338
**Age, years**	55 (47-67)	59 (52-67)	53 (46-64)	0.049
**Disease duration (months)**	NA	24 (4-66)	12 (5-48)	0.294
**Hypertension, n (%)**	NA	7 (13.21)	20 (20.2)	0.282
**Diabetes mellitus, n (%)**	NA	1 (1.87)	2 (2.02)	1.000
**Clinical manifestations, n (%)**				
**Oral dryness**	NA	38 (71.7)	64 (64.65)	0.378
**Ocular dryness**	NA	31 (58.49)	52 (52.53)	0.481
**Rampant caries**	NA	12 (22.64)	21 (21.21)	0.839
**Fatigue**	NA	10 (18.87)	26 (26.26)	0.307
**Raynaud’s phenomenon**	NA	9 (16.98)	3 (3.03)	0.006
**Sclerodactyly**	NA	0 (0)	0 (0)	1.000
**Cutaneous involvement**	NA	8 (15.09)	13 (13.13)	0.738
**Articular involvement**	NA	19 (35.85)	29 (29.29)	0.407
**Nervous system involvement**	NA	3 (5.66)	9 (9.09)	0.666
**Digestive involvement**	NA	9 (16.98)	11 (11.11)	0.308
**Pulmonary involvement**	NA	4 (7.55)	8 (8.08)	1.000
**Left ventricular diastolic dysfunction**	NA	27 (50.94)	33 (33.33)	0.034
**Electrocardiographic abnormalities**	NA	14 (26.42)	18 (18.18)	0.235
**Renal involvement**	NA	2 (3.77)	5 (5.05)	1.000
**Haematological involvement**	NA	9 (16.98)	38 (38.38)	0.007
**Anemia**	NA	5 (9.43)	29 (29.29)	0.005
**Leukopenia**	NA	7 (13.21)	31 (31.31)	0.014
**Neutropenia**	NA	4 (7.55)	22 (22.22)	0.022
**Lymphopenia**	NA	8 (15.09)	22 (22.22)	0.293
**Thrombocytopenia**	NA	2 (3.77)	13 (13.13)	0.065
**Personal history of cancer, n (%)**	NA	6 (11.32)	13 (13.13)	0.748
**Lung cancer**	NA	3 (5.66)	2 (2.02)	0.343
**Breast cancer**	NA	2 (3.77)	2 (2.02)	0.611
**Digestive tract malignant tumors**	NA	1 (1.89)	3 (3.03)	1.000
**Thyroid cancer**	NA	0 (0)	6 (6.06)	0.092
**Renal malignant tumors**	NA	0 (0)	2 (2.02)	0.543
**Other autoimmune diseases, n (%)**	NA	7 (13.21)	14 (14.14)	0.874
**Autoimmune hepatitis**	NA	3 (5.66)	7 (7.07)	1.000
**Primary biliary cholangitis**	NA	2 (3.77)	0 (0)	0.120
**Autoimmune thyroid disease**	NA	2 (3.77)	9 (9.09)	0.380

NA: not applicable

### Routine blood tests, biochemical and immunological parameters, and Echocardiography

The routine blood tests, biochemical, immunological parameters, and echocardiographic results of the enrolled subjects are summarized in Table 2. There were no significant differences in ALT, ALP, GGT, C4 levels, and monocyte counts among the three groups. However, significant differences were identified in WBC, NE, LC, RBC, Hb, PLT, GLB, ALB, AST, LDH, IgG, IgM, IgA, and C3 levels (P < 0.05 for all). Post hoc analysis indicated that both the ACA-positive and SSA-positive groups had significantly lower WBC, NE, LC, and PLT, alongside increased AST levels compared to healthy controls (P < 0.05 for all). The SSA-positive group also displayed lower RBC compared to healthy controls, as well as lower RBC and ALB levels than the ACA-positive group (P < 0.05 for all). Furthermore, ACA-positive patients demonstrated significantly increased LDH levels compared to both healthy controls and the SSA-positive group (P < 0.05 for both). In contrast to the healthy controls, the SSA-positive group exhibited significantly lower C3 levels and concurrently higher IgG and IgA levels, which were notably more pronounced than those in the ACA-positive group (P < 0.05 for all). Although IgM levels in the ACA-positive group were significantly higher than those in healthy controls (P < 0.05), the levels in the ACA-positive group were also higher but did not reach statistical significance compared to the SSA-positive group (P = 0.078). Consistent with previous findings, there were no significant differences in the proportions of abnormal C4 and IgM across the groups (P > 0.05 for both); however, variations in IgG, IgA, C3, and RF abnormalities were noted, with the SSA-positive group exhibiting a significantly higher prevalence of IgG, IgA, and RF abnormalities compared to both ACA-positive and healthy control groups (P < 0.05 for all), and no significant difference between the ACA-positive and healthy controls (P > 0.05 for all). Both ACA-positive and SSA-positive groups had significantly higher proportions of C3 abnormalities compared to healthy controls (P < 0.05 for both). In terms of autoantibodies, the positivity rate for ANA and AMA-M2 was significantly higher in the ACA-positive group than in the SSA-positive group (P < 0.05 for both), while all autoantibodies were negative in the healthy control group. Among SSA-positive patients, the positive rates for anti-SSA/Ro52, anti-SSA/Ro60, and anti-SSB/La antibodies were 88 cases (88.89%), 80 cases (80.81%), and 38 cases (38.38%), respectively. All other autoantibodies, including anti-nRNP, anti-Sm, anti-Scl-70, anti-PM-Scl, anti-Jo-1, anti-PCNA, anti-dsDNA, anti-histones, anti-nucleosomes, and anti-RibP were negative in all pSS patients. Echocardiographically, there were no significant disparities in IVST, LAD, or LVEF between the two patient groups (P > 0.05 for all); however, the LVEDD was notably elevated in the ACA-positive patients compared to the SSA-positive patients (P < 0.05).

**Table 2:** Routine blood tests, biochemical, and immunological parameters.

Parameters	Healthy controls (n = 105)	ACA-positive (n = 53)	SSA-positive (n = 99)	P-value
**WBC (×10** ^**9**^ **/L)**	5.6 (4.8-6.4)	4.6 (3.8-5.7)***	4.2 (3.3-5.1)***	<0.001
**NE (×10** ^**9**^ **/L)**	3.1 (2.5-3.6)	2.4 (1.9-3.0)**	2.3 (1.7-3.0)***	<0.001
**LC (×10** ^**9**^ **/L)**	1.9 (1.6-2.3)	1.6 (1.2-1.8)***	1.4 (1.1-1.7)***	<0.001
**Mono (×10** ^**9**^ **/L)**	0.4 (0.3-0.5)	0.4 (0.3-0.5)	0.4 (0.3-0.4)	0.082
**RBC (×10** ^**12**^ **/L)**	4.35 ± 0.45	4.17 ± 0.46	3.94 ± 0.54***, ^	<0.001
**Hb, g/L**	130 ± 14	124 ± 14	118 ± 17***, ^	<0.001
**PLT (×10** ^**12**^ **/L)**	227 ± 47	194 ± 53**	192 ± 67***	<0.001
**ALB, g/L**	43.7 ± 3.3	42.0 ± 3.7	40.1 ± 4.2***, ^	<0.001
**GLB, g/L**	25.1 (22.9-27.5)	24.8 (22.2-28.0)	29.5 (25.8-33.5)***, ^	<0.001
**ALT, U/L**	17 (13-24)	19 (13-25)	19 (13-30)	0.371
**AST, U/L**	19 (16-23)	23 (19-30)**	23 (18-29)***	<0.001
**ALP, U/L**	71 (57-84)	69 (54-86)	68 (56-86)	0.934
**GGT, U/L**	17 (12-27)	19 (12-44)	17 (11-31)	0.313
**LDH, mg/L**	168 (154-191)	184 (162-211)*	168 (147 -198)^	0.030
**IgG, g/L**	12.5 (10.5-14.4)	12.7 (10.7-15.0)	17.0 (15.1-21.1)***, ^	<0.001
**IgM, g/L**	1.12 (0.75-1.48)	1.51 (0.97-1.95)**	1.10 (0.91-1.57)	0.011
**IgA, g/L**	2.36 (1.79-3.14)	2.27 (1.77-3.21)	3.03 (2.48-4.08)***, ^	<0.001
**C3, mg/dL**	88.8 (80.1-102.0)	86.9 (73.6-99.8)	82.8 (71.7-98.0)*	0.015
**C4, mg/dL**	19.8 (16.0-22.4)	19.3 (16.0-25.4)	18.1 (15.9-22.7)	0.430
**Hyper-IgG, n (%)**	3 (2.86)	4 (7.55)	47 (47.47)***, ^	<0.001
**Hyper-IgM, n (%)**	2 (1.9)	2 (3.77)	8 (8.08)	0.102
**Hyper-IgA, n (%)**	1 (0.95)	2 (3.77)	22 (22.22)***, ^	<0.001
**Lower-C3, n (%)**	5 (4.76)	11 (20.75)**	22 (22.22)***	0.001
**Lower-C4, n (%)**	1 (0.95)	1 (1.89)	2 (2.02)	0.839
**RF > 20 U/mL, n (%)**a	2 (1.9)	2 (3.77)	42 (42.42)***, ^	<0.001
**ANA titres ≥1:320, n (%)**b	0 (0)	46 (86.79)***	71 (71.72)***, ^	<0.001
**AMA-M2, n (%)**	0 (0)	14 (26.42)***	7 (7.07)**, ^	<0.001
**ACA, n (%)**	0 (0)	53 (100)***	0 (0)^	<0.001
**Anti-SSA/Ro, n (%)**	0 (0)	0 (0)	99 (100)***, ^	<0.001
**Anti-Ro52, n (%)**	0 (0)	0 (0)	88 (88.89)***, ^	<0.001
**Anti-Ro60, n (%)**	0 (0)	0 (0)	80 (80.81)***, ^	<0.001
**Anti-SSB/La, n (%)**	0 (0)	0 (0)	38 (38.38)***, ^	<0.001
**LVEDD, mm**	NA	45 (43-46)	44 (42-46)^	0.048
**IVST, mm**	NA	9.3 (8.4-10)	9.1 (8.4-9.6)	0.274
**LAD, mm**	NA	31 (29-32)	30 (29-32)	0.610
**LVEF, %**	NA	66 (63-69)	66 (65-68)	0.870

The indicators with a positivity rate of zero in all three groups are not listed in the table; apositive RF > 20 IU/ml; bpositive for ANA titres ≥ 1:320; *, P < 0.05 vs. Healthy controls; **, P < 0.01 vs. Healthy controls; ***, P < 0.001 vs. Healthy controls; ^, P < 0.05 vs. ACA-positive. NA: not applicable.

### Lymphocyte subsets in peripheral blood

**Fig 1:**
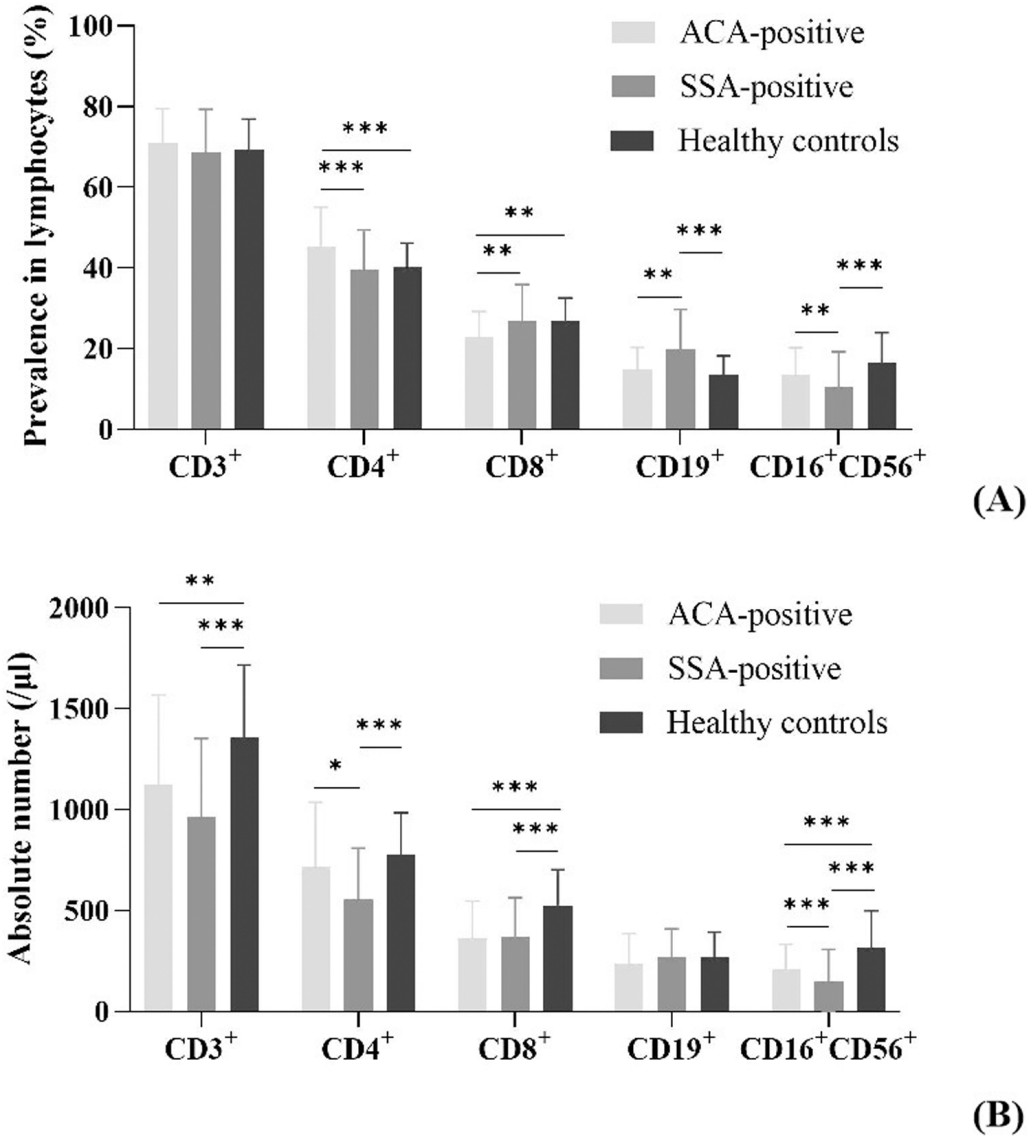
The prevalence and absolute counts of peripheral blood Lymphocyte subsets. A: Prevalence of T cells (CD3^+^, CD4^+^, CD8^+^), B cells (CD19^+^), and NK cells (CD16^+^CD56^+^) in the lymphocytes of healthy controls and pSS patients. B: Absolute numbers of T cells, B cells, and NK cells in the lymphocytes of healthy controls and pSS patients. *P < 0.05; **P < 0.01; ***P < 0.001.

The distribution of T cells (CD3^+^, CD4^+^, CD8^+^), B cells (CD19^+^) and NK cells (CD16^+^CD56^+^) across the three groups is showed in Fig 1. No significant differences were found in the prevalence of CD3^+^ T cells or the absolute counts of B cells (P > 0.05). Compared to the healthy control group, both the ACA-positive and SSA-positive groups had significantly lower absolute counts of CD3^+^ and CD8^+^ T cells (P < 0.01). The SSA-positive group had a significantly higher proportion of B cells than the ACA-positive group and healthy controls (P < 0.001), with no significant difference between the ACA-positive group and healthy controls (P > 0.05). Despite this, the absolute counts of B cells were similar across all groups (P > 0.05). The SSA-positive group exhibited a significantly lower proportion of NK cells than the ACA-positive and healthy control groups (P < 0.01). The healthy control group had a higher absolute count of NK cells than the ACA-positive group (P < 0.001), which, in turn, had a higher count than the SSA-positive group (P < 0.001). The ACA-positive group had a higher proportion of CD4^+^ T cells than the SSA-positive group and healthy controls (P < 0.001), and a lower proportion of CD8^+^ T cells (P < 0.01). The CD4^+^ T cell count in the SSA-positive group was significantly lower than in the ACA-positive group and healthy controls (P < 0.05), with no significant difference between the ACA-positive and healthy control groups (P > 0.05).

### The associations between immune markers, biochemical parameters and lymphocyte subsets

The associations between immune markers, biochemical parameters, and lymphocyte subpopulations in the ACA-positive group are presented in Tables 3 and 4. Serum IgM levels significantly correlated with CD3^+^ T cell proportions and CD16^+^CD56^+^ NK cell proportions and counts, while serum IgA levels were positively associated with T cells counts(CD3^+^, CD4^+^, CD8^+^). Serum C3 levels positively correlated with the absolute counts of T cells (CD3^+^, CD4^+^, CD8^+^), CD19^+^ B cells, and CD16^+^CD56^+^ NK cells, and serum C4 levels were only positively related to the absolute count of CD8^+^ T cells. After adjusting for age and sex, IgM levels remained correlated with CD16^+^CD56^+^ NK cell proportions (r = 0.246, P = 0.082) and counts (r = 0.264, P = 0.061), and IgA levels significantly correlated with T cell counts (CD3^+^: r = 0.399, CD4^+^: r = 0.361, CD8^+^: r = 0.302; P < 0.05 for all). Serum C3 levels significantly correlated with T cells (CD3^+^: r = 0.541, CD4^+^: r = 0.442, CD8^+^: r = 0.430; P < 0.05 for all), CD19^+^ B cells (r = 0.375, P < 0.01), and CD16^+^CD56^+^ NK cells (r = 0.296, P < 0.05), and serum C4 levels were significantly associated with CD8^+^ T cell counts (r = 0.331, P < 0.05). Additionally, biochemical parameters such as TP, GLB, and LDH were significantly associated with CD16^+^CD56^+^ NK cell proportions and counts. ALT levels were associated with CD8^+^ T cell proportions, AST levels with CD3^+^ T cell proportions, and ALP levels with the proportions of CD3^+^ and CD4^+^ T cell. After adjusting for age and gender, significant correlations persisted between TP and LDH levels and CD16^+^CD56^+^ NK cell proportions (TP: r = 0.288; LDH: r = 0.356; P < 0.05 for both), and between TP, GLB, and LDH levels and CD16^+^CD56^+^ NK cell absolute counts (TP: r = 0.341; GLB: r = 0.364; LDH: r = 0.305; P < 0.05 for all).

**Table 3:** Correlation between immune function indicators and Lymphocyte subsets in pSS patients.

Parameters	IgG		IgM		IgA		C3		C4	
	r	P	r	P	r	P	r	P	r	P
**ACA-positive group**										
**% CD3** ^**+**^	-0.201	0.148	-0.313	**0.022**	-0.070	0.621	0.089	0.524	0.087	0.536
**% CD4** ^**+**^	-0.104	0.460	-0.118	0.399	-0.048	0.621	-0.017	0.905	-0.156	0.266
**% CD8** ^**+**^	-0.033	0.816	-0.264	0.056	-0.041	0.770	0.117	0.405	0.234	0.091
**% CD19** ^**+**^	0.041	0.771	-0.003	0.985	0.014	0.919	-0.060	0.668	-0.158	0.260
**% CD16** ^** + **^ **CD56** ^**+**^	0.208	0.135	0.354	**0.009**	0.071	0.613	0.042	0.765	0.102	0.469
**CD3** ^** +**^ ** count**	0.087	0.536	0.081	0.562	0.332	**0.015**	0.494	**<0.001**	0.228	0.100
**CD4** ^** +**^ ** count**	0.023	0.871	0.173	0.215	0.281	**0.041**	0.344	**0.012**	0.102	0.469
**CD8** ^** +**^ ** count**	0.083	0.555	-0.056	0.692	0.318	**0.020**	0.479	**<0.001**	0.354	**0.009**
**CD19** ^** +**^ ** count**	0.135	0.337	0.076	0.587	0.256	0.064	0.278	**0.044**	0.030	0.832
**CD16** ^** + **^ **CD56** ^** +**^ ** count**	0.219	0.115	0.354	**0.009**	0.248	0.074	0.326	**0.017**	0.229	0.100
**SSA-positive group**										
**% CD3** ^**+**^	-0.181	0.073	-0.085	0.402	-0.094	0.355	0.056	0.584	-0.077	0.450
**% CD4** ^**+**^	-0.215	**0.033**	-0.115	0.259	-0.202	**0.045**	0.008	0.936	0.045	0.656
**% CD8** ^**+**^	-0.005	0.961	-0.052	0.613	0.093	0.361	0.100	0.325	-0.045	0.655
**% CD19** ^**+**^	0.152	0.134	-0.042	0.678	0.076	0.457	-0.025	0.807	0.010	0.924
**% CD16** ^** + **^ **CD56** ^**+**^	0.006	0.949	0.054	0.594	-0.037	0.717	0.089	0.382	0.276	**0.006**
**CD3** ^** +**^ ** count**	-0.233	**0.020**	0.083	0.414	0.071	0.484	0.184	0.069	0.100	0.323
**CD4** ^** +**^ ** count**	-0.274	**0.006**	0.040	0.696	0.027	0.793	0.173	0.087	0.146	0.149
**CD8** ^** +**^ ** count**	-0.110	0.277	0.101	0.321	0.163	0.108	0.110	0.279	-0.006	0.956
**CD19** ^** +**^ ** count**	0.048	0.639	-0.022	0.832	0.232	**0.021**	0.177	0.079	0.162	0.109
**CD16** ^** + **^ **CD56** ^** +**^ ** count**	-0.097	0.340	0.103	0.312	-0.029	0.778	0.082	0.419	0.283	**0.005**

^a^Bold values indicate P < 0.05.

**Table 4:** Correlation between biochemical indicators and Lymphocyte subsets in pSS patients.

Parameters	ALB		GLB		ALT		AST		ALP		GGT		LDH	
	r	P	r	P	r	P	r	P	r	P	r	P	r	P
**ACA-positive group**														
**% CD3** ^**+**^	-0.055	0.697	-0.226	0.103	-0.257	0.063	-0.346	**0.011**	-0.335	**0.014**	-0.156	0.263	-0.268	0.052
**% CD4** ^**+**^	-0.154	0.270	-0.191	0.171	0.096	0.494	-0.034	0.807	-0.325	**0.018**	-0.165	0.238	-0.198	0.154
**% CD8** ^**+**^	0.151	0.279	-0.028	0.843	-0.274	**0.047**	-0.258	0.062	-0.101	0.474	0.006	0.965	-0.057	0.686
**% CD19** ^**+**^	-0.094	0.501	-0.032	0.820	0.178	0.202	0.234	0.092	0.095	0.498	0.074	0.600	-0.081	0.565
**% CD16** ^** + **^ **CD56** ^**+**^	0.262	0.058	0.284	**0.039**	0.174	0.213	0.257	0.063	0.230	0.098	0.120	0.394	0.313	**0.023**
**CD3** ^** +**^ ** count**	-0.015	0.914	0.238	0.087	0.053	0.704	-0.004	0.979	-0.020	0.884	0.164	0.242	-0.022	0.876
**CD4** ^** +**^ ** count**	-0.114	0.417	0.100	0.476	0.124	0.377	0.023	0.869	-0.147	0.292	0.081	0.566	-0.049	0.728
**CD8** ^** +**^ ** count**	0.043	0.761	0.166	0.235	-0.115	0.414	-0.134	0.339	-0.065	0.644	0.111	0.427	-0.044	0.755
**CD19** ^** +**^ ** count**	-0.136	0.332	0.086	0.540	0.176	0.209	0.185	0.185	0.068	0.628	0.109	0.437	-0.026	0.851
**CD16** ^** + **^ **CD56** ^** +**^ ** count**	0.210	0.131	0.377	**0.005**	0.149	0.286	0.224	0.107	0.259	0.062	0.152	0.278	0.306	**0.026**
**SSA-positive group**														
**% CD3** ^**+**^	-0.104	0.305	-0.264	**0.008**	-0.034	0.739	-0.107	0.292	0.029	0.774	0.086	0.398	-0.267	**0.007**
**% CD4** ^**+**^	0.021	0.838	-0.336	**<0.001**	0.075	0.463	-0.010	0.920	-0.013	0.899	0.137	0.177	-0.024	0.811
**% CD8** ^**+**^	-0.077	0.448	0060	0.556	-0.036	0.727	-0.097	0.337	0.054	0.597	0.039	0.703	-0.279	**0.005**
**% CD19** ^**+**^	0.015	0.882	0.114	0.260	-0.022	0.826	-0.055	0.587	-0.093	0.362	-0.104	0.304	0.120	0.237
**% CD16** ^** + **^ **CD56** ^**+**^	0.114	0.262	0.180	0.075	0.008	0.939	0.087	0.392	0.114	0.261	0.076	0.455	0.139	0.172
**CD3** ^** +**^ ** count**	0.241	**0.016**	-0.230	**0.022**	0.002	0.987	-0.037	0.720	0.193	0.056	0.167	0.099	-0.215	**0.032**
**CD4** ^** +**^ ** count**	0.256	**0.010**	-0.294	**0.003**	0.033	0.749	-0.024	0.815	0.165	0.103	0.160	0.113	-0.132	0.191
**CD8** ^** +**^ ** count**	0.196	0.052	-0.079	0.436	0.000	0.996	-0.040	0.697	0.168	0.097	0.142	0.160	-0.234	**0.020**
**CD19** ^** +**^ ** count**	0.134	0.185	0.074	0.465	-0.008	0.935	-0.023	0.822	0.096	0.342	0.054	0.597	0.104	0.304
**CD16 + CD56 + count**	0.321	**0.001**	-0.040	0.695	-0.014	0.890	0.024	0.814	0.128	0.205	0.035	0.730	0.055	0.589

^a^Bold values indicate P < 0.05.

The associations between immune markers, biochemical parameters, and lymphocyte subpopulations in the SSA-positive group are presented in Tables 3 and 4. Serum IgG levels were significantly associated with CD4^+^ T cell proportion and CD3^+^ and CD4^+^ T cell counts. IgA levels correlated with CD4^+^ T cell proportion and CD19^+^ B cell counts, while C4 levels were linked to CD16^+^CD56^+^ NK cell proportion and counts. After adjusting for age and gender, significant correlations persisted between IgG levels and CD4^+^ T cell proportion (r = -0.204, P < 0.05) and CD3^+^ (r = -0.204, P < 0.05) and CD4^+^ (r = -0.276, P < 0.01) T cell counts. Additionally, biochemical parameters such as ALB, GLB, and LDH were associated with lymphocyte subpopulations. ALB levels were significantly associated with the proportions and absolute counts of CD16^+^CD56^+^ NK cells, CD3^+^ T cells, and CD4^+^ T cells, respectively. GLB levels correlated with CD3^+^ and CD4^+^ T cell proportion and counts, and LDH levels with CD3^+^ and CD8^+^ T cell proportion and counts. Adjusting for age and gender maintained significant correlations between ALB levels and the absolute counts of CD16^+^CD56^+^ NK cells (r = 0.260, P = 0.01), CD3^+^ T cells (r = 0.250, P < 0.05), and CD4^+^ T cells (r = 0.273, P < 0.01). GLB levels significantly correlated with CD4^+^ T cell proportion (r = -0.317, P < 0.01) and CD3^+^ (r = -0.203, P < 0.05) and CD4^+^ (r = -0.296, P < 0.01) T cell counts. LDH levels were significantly associated with both the proportion (CD3^+^: r = -0.358; CD8^+^: r = -0.278; P < 0.01 for both) and counts (CD3^+^: r = -0.293; CD8^+^: r = -0.314; P < 0.01 for both) of T cells.

### Multivariate stepwise regression analysis on relationships between immune markers, biochemical parameters and lymphocyte subsets

Multivariate regression analysis was performed to evaluate the correlations between immune markers, biochemical parameters, and lymphocyte subsets (Table 5). In the ACA-positive group, serum levels of IgG, IgM, and GLB were independently correlated with the absolute count of CD16^+^CD56^+^ NK cells (IgG: β = 0.296; IgM: β = 0.399; GLB: β = 0.407; P < 0.05 for all), while serum LDH levels were independently associated with the percentage of CD16^+^CD56^+^ NK cells (β = 0.357, P < 0.01). Serum IgA and C3 levels showed independent correlations with the count of CD3^+^ T cells (IgA: β = 0.394; C3: β = 0.531; P < 0.01 for both). Serum C4 levels were independently associated with the absolute count of CD8^+^ T cells (β = 0.326, P < 0.05). Additionally, in the SSA-positive group, serum levels of IgG and ALB were independently correlated with the absolute count of CD4^+^ T cells (IgG: β = -0.278; ALB: β = 0.256; P < 0.05 for both). Serum GLB levels were independently associated with the percentage of CD4^+^ T cells (β = -0.296; P < 0.01). Serum LDH levels were independently correlated with the percentage of CD3^+^ T cells (β = -0.367, P < 0.001).

**Table 5:** Correlation between immune function indicators and Lymphocyte subsets in pSS patients.

Groups	Marker	Lymphocyte Parameter	-coefficient	95% CI	P value
**ACA-positive group**	IgG	NK cells count	0.296	0.001, 0.012	0.031
	IgM	NK cells count	0.399	0.001, 0.004	0.003
	C3	CD3^ +^ count	0.531	0.011, 0.028	<0.001
	C4	CD8^ +^ count	0.326	0.003, 0.027	0.017
	GLB	NK cells count	0.407	0.005, 0.023	0.002
	LDH	% NK cells	0.357	0.547, 3.598	0.009
**SSA-positive group**	IgG	CD4^ +^ count	-0.278	-0.013, -0.002	0.005
	ALB	CD4^ +^ count	0.256	0.001, 0.008	0.010
	GLB	% CD4^+^	-0.296	-0.379, -0.080	0.003
	LDH	% CD3^+^	-0.367	-2.005, -0.650	<0.001

## Discussion

Our study found that ACA-positive pSS patients were significantly older than those who were SSA-positive. The two groups differed in the prevalence of Raynaud’s phenomenon, hematological involvement and left ventricular diastolic dysfunction, with the first two clinical manifestations aligning with findings from previous studies [8–10,14]. Notably, laboratory tests revealed variations in immune-related parameters among the three groups, indicating differences in pathogenic mechanisms associated with distinct autoantibodies. Continuous monitoring of these markers may yield valuable insights into disease progression and treatment efficacy.

The anti-centromere antibody (ACA) is widely recognized as a specific biomarker for systemic sclerosis (SSc), a disorder characterized by microvascular abnormalities and the progression of tissue fibrosis [15,16]. These abnormalities in microvasculature are frequently associated with the onset of Raynaud’s phenomenon [17]. In our study, the prevalence of Raynaud’s phenomenon was significantly higher among pSS patients with positive ACA than among those with positive SSA/Ro, consistent with recent studies [10,18]. Furthermore, our laboratory data from pSS patients with positive ACA showed significantly higher levels of lactate dehydrogenase and left ventricular end-diastolic diameter, and a greater incidence of left ventricular diastolic dysfunction. This suggests that ACA-positive patients with pSS require heightened attention and monitoring for cardiovascular-related diseases compared to those with SSA positivity. Previous research has indicated that Raynaud’s phenomenon is a potent risk factor associated with the onset of pulmonary hypertension and left-ventricular regional dysfunction in patients with autoimmune disorders [19,20]. Our research also indirectly supports this observation. Since left ventricular diastolic dysfunction is one of the mechanisms leading to heart failure in patients with rheumatic diseases, and heart failure is a primary cause of mortality in these patients, continuous monitoring of the cardiovascular health of pSS patients, particularly those with ACA, is crucial for prognosis assessment.

The anti-SSA/Ro antibody, a common autoimmune marker, can be detected in various diseases, including systemic lupus erythematosus (SLE), Sjögren’s syndrome (SS), systemic sclerosis (SSc), rheumatoid arthritis (RA), autoimmune liver disease, and myositis [21,22]. However, anti-SSA/Ro antibodies are considered one of the specific diagnostic indicators in pSS patients [11]. Studies have shown that patients with anti-SSA/Ro antibodies are more likely to have other autoimmune antibodies, such as rheumatoid factor (RF) [23], and they exhibit significantly increased risk factors for involvement of other organs [24,25]. Consistently, nearly half of the anti-SSA/Ro-positive pSS patients in our study tested positive for rheumatoid factor, with high titers > 80 U/mL, although specific titers are not shown here. This finding suggests an increased likelihood of concurrent rheumatoid arthritis. Additionally, compared to the ACA-positive group, patients with SSA-positive pSS exhibited a significantly higher rate of hematological involvement, encompassing anemia, leukopenia, and neutropenia, than those in the ACA-positive group (P < 0.05). This phenomenon has been reported in numerous previous studies as well [10,14,18]. These results suggest that anti-SSA/Ro antibodies may have a considerable systemic impact compared to ACA in pSS patients. Therefore, the systemic clinical symptoms influenced by anti-SSA/Ro antibodies may also contribute to the observation that SSA-positive pSS patients tend to be diagnosed at a significantly younger age than their ACA-positive counterparts.

Whole blood lymphocyte subsets are closely associated with the disease activity of Sjögren’s syndrome [26]. Significant differences in these parameters were observed between the two groups of pSS patients, indicating disease heterogeneity among different pSS subtypes. CD19^+^ B cells are critical in the pathophysiology of Sjögren’s syndrome due to their overactivation, which leads to the production of various autoantibodies. These autoantibodies manifest as abnormalities in immunoglobulin levels and may target and damage autologous tissues, contributing to the clinical symptoms of the disease [27]. As reported in previous research [10], the current study shows that patients with pSS who are positive for SSA, in contrast to those with positive ACA or healthy subjects, had a significantly higher proportion of CD19^+^ B cells, along with increased concentrations of globulin (GLB), IgG, IgA, and RF. This suggests that SSA-positive patients may exhibit a more severe disease phenotype, characterized by heightened B cell activity and autoimmune responses. Consistently, research by Seror et al. [28] demonstrated that rituximab can significantly alleviate clinical manifestations in patients with systemic involvement while reducing rheumatoid factor, γ-globulin, and β2-microglobulin levels. However, clinical studies have shown that not all patients with pSS respond to this treatment [29], suggesting that the disease may involve complex autoimmune mechanisms. Other immune cells, such as T cells and NK cells, likely play significant roles in the pathology, indicating that B cell depletion alone may not effectively modulate the immune response. In our study, after adjusting for age and gender, we found that serum levels of globulins (GLB), albumin (ALB), and IgG in SSA-positive pSS patients were significantly correlated with CD4^+^ T cells, and multivariate regression analysis further demonstrated that these parameters were independently associated with CD4^+^ T cells. These findings underscore the critical role of interactions between CD4^+^ T cells and CD19^+^ B cells in the pathophysiology of Sjögren’s syndrome. Specifically, a negative feedback mechanism may exist between CD4^+^ T cells and CD19^+^ B cells, where CD4^+^ T cells secrete various cytokines (e.g., interleukin-21, interleukin-17, and IFN-γ) [30] that lead to the abnormal activation and differentiation of B cells, resulting in the production of large amounts of autoantibodies, abnormal immunoglobulins, and additional cytokines. This overproduction (e.g., interleukin-6, interleukin-10, and interleukin-35) can feedback to the body, subsequently causing a decrease in the absolute count of other lymphocyte populations [31]. In our comparative study of Sjögren’s syndrome patients and healthy controls, we observed no significant difference in the absolute count of B cells; however, the absolute counts of other lymphocyte subsets were markedly reduced in the Sjögren’s syndrome patients, which further confirms the intricate relationship between these immune cells.

CD8^+^ T cells also play a important role in the pathology of Sjögren’s syndrome, exerting cytotoxic effects that target glandular cells and result in glandular damage and dysfunction [32].Previous studies have shown increased T cell infiltration in the salivary glands of patients with Sjögren’s syndrome, primarily consisting of CD4^+^ and CD8^+^ T cells [33,34], with the latter rarely undergoing local proliferation during disease progression but being readily recruited and differentiated from peripheral sources [35]. This suggests that cell migration may be one of contributing factor to the decreased absolute counts of CD8^+^ T cells in the peripheral blood of these patients. Moreover, after adjusting for age and sex, we found that the absolute count of peripheral blood CD8^+^ T cells in ACA-positive pSS patients was significantly correlated with complement levels C3 and C4. Multivariate regression analysis further indicated that the absolute count of CD8^+^ T cells independently correlated with complement C4 levels. These results indicate that changes in the absolute count of CD8^+^ T cells in ACA-positive pSS patients may be directly associated with complement activation status, which could promote CD8^+^ T cell activation or proliferation. Notably, numerous clinical studies have demonstrated a strong association between complement levels and disease activity in autoimmune disorders [14,36,37], suggesting that CD8^+^ T cells may serve as potential biomarkers for predicting disease activity in Sjögren’s syndrome. Interestingly, no correlation was found between CD8^+^ T cells and complement levels in patients with SSA-positive primary Sjögren’s syndrome. This suggests that the immune responses and pathological mechanisms may differ between the two patient groups. Antibodies against SSA/Ro might trigger distinct types of immune responses, leading to variations in complement activation patterns and lymphocyte responses.

CD16^+^CD56^+^ natural killer (NK) cells play a complex and pivotal role in autoimmune diseases. As crucial immune cells, they possess the ability to recognize and eliminate infected or tumor cells, while also contributing to the regulation of immune responses. Our study found that the absolute count of CD16^+^CD56^+^ NK cells was significantly lower in both pSS groups compared to healthy controls, which may disrupt immune surveillance and contribute to the higher incidence of tumors in patients with Sjögren’s syndrome [38]. In our study, the elevated malignancy incidence rates in both SS patients, with 11.32% in the ACA-positive group and 14.13% in the SSA-positive group, further support this observation. Additionally, previous studies have indicated that patients with Sjögren’s syndrome are more likely to develop autoimmune liver diseases, such as autoimmune hepatitis (AIH) and primary biliary cholangitis (PBC), compared to those with other autoimmune disorders [39,40]. The present study compared the incidence of autoimmune liver disease between two cohorts of pSS patients, concurrently analyzing serum biochemical and immunological parameters, as well as the presence of anti-mitochondrial M2 antibody (AMA-M2). No significant disparities were found in liver enzyme levels or the prevalence of autoimmune liver diseases between the two pSS cohorts. Notably, the AMA-M2 positivity rate was substantially higher in ACA-positive pSS patients (26.42%) than in SSA-positive patients (7.07%, P < 0.05). Research indicates that in the initial phase of PBC, liver function often remains normal or slightly elevated, with the presence of ANA, particularly those with centromere positivity, serving as a potential early indicator [41]. In our study, the ACA-positive subgroup exhibited elevated IgM levels. Correlation and multiple regression analyses revealed an independent positive association between CD16^+^CD56^+^ NK cell counts and IgM levels in ACA-positive pSS patients, despite the overall reduction in NK cell counts in pSS. This suggests that the remaining NK cells may modulate IgM production or maintain immune homeostasis in ACA-positive pSS patients. Since AMA-M2 is a hallmark antibody of primary biliary cholangitis (PBC), and PBC patients typically show elevated IgM levels [41], ACA-positive pSS patients may be at an enhanced risk for developing PBC, potentially due to dysregulated CD16^+^CD56^+^ NK cell activation.

This retrospective study has several limitations. First, due to the chronic nature of Sjögren’s syndrome, which typically has a prolonged course, we excluded patients who had received treatments such as corticosteroids or immunosuppressants to minimize their impact on the results. As a result, the relatively small sample size may reduce the statistical power of our analysis. Additionally, the study was conducted at a single center, which limited the number of enrolled patients and may introduce selection bias. Furthermore, as this study relied on data from medical records, despite thorough verification and consistency checks, issues of incomplete or inaccurate information may still exist, potentially introducing biases and confounding factors. Therefore, the conclusions of this study require validation through prospective, multicenter studies or experimental research.

## Conclusions

This retrospective study elucidates the distinct clinical and laboratory characteristics of pSS patients stratified by autoantibody status. ACA-positive pSS patients were significantly older and exhibited higher prevalence rates of Raynaud’s phenomenon and left ventricular diastolic dysfunction, while SSA-positive patients demonstrated more pronounced hematological abnormalities. Laboratory analyses revealed significant differences in lymphocyte subsets, biochemical parameters, and immune markers. SSA-positive patients had elevated levels of GLB, IgG, IgA, RF, and CD19^+^ B cells, indicating enhanced autoimmune responses. In contrast, ACA-positive individuals showed increased levels of IgM, LDH and LVEDD, with a notably higher rate of AMA-M2 positivity, suggesting a possible link to cardiovascular complications and primary biliary cholangitis. Correlation and multiple regression analyses highlighted the importance of T cell-mediated immunity in SSA-positive patients, while indicating the crucial role of NK cells in the pathophysiology of ACA-positive pSS. These insights underscore the need for tailored monitoring and management strategies for different pSS subtypes, which may inform future therapeutic approaches.

## Supporting information

S1 Data Anonymized clinical datasets (patient demographics, medical imaging, and laboratory test records). (XLS)
